# Isolation and Characterization of Swinepox Virus from Outbreak in Russia

**DOI:** 10.3390/ani13111786

**Published:** 2023-05-27

**Authors:** Andrey Koltsov, Mikhail Sukher, Natalia Kholod, Sanzhi Namsrayn, Sodnom Tsybanov, Galina Koltsova

**Affiliations:** Federal Research Centre for Virology and Microbiology, Academician Bakoulov Street 1, 601125 Volginsky, Vladimir Region, Russia; kolcov.andrew@gmail.com (A.K.); suhermail@mail.ru (M.S.); natkholod@yandex.ru (N.K.); namsrayn.szh@gmail.com (S.N.); cybanov@mail.ru (S.T.)

**Keywords:** swinepox virus, outbreak, phylogeny, Russian isolate

## Abstract

**Simple Summary:**

Pork is one of the most important types of sources of protein in the human diet. Swine diseases cause major economic losses to the global pig industry. Early diagnosis and prevention of diseases are fundamental factors for maintaining the health of animals on pig farms. The present article is the first report on the successful isolation of the swinepox virus in Russia. The results of a laboratory experiment on animals revealed a low pathogenicity of the Russian isolate for pigs. We suggest that this isolate can be used as a viral vector for the development of vaccines for animals. In addition, we have demonstrated for the first time that nasal and oral swabs can be used for PCR diagnosis of the disease and for swinepox virus isolation.

**Abstract:**

Swinepox virus (SWPV) is the only member of the *Suipoxvirus* genus of the *Poxviridae* family and is an etiologic agent of a worldwide disease specific for domestic and wild pigs. SWPV outbreaks are sporadically recorded in different regions of Russia. In 2013, an outbreak of the disease causing skin lesions was registered on a pig farm in Russia. The presence of SWPV in the scab samples was assessed by in-house real-time PCR, reference PCR amplification, and nucleotide sequencing of the viral late transcription factor-3 (VLTF-3) gene and was then confirmed by virus isolation. Thus, the in-house real-time PCR proposed in this study could serve as a useful tool for the rapid specific detection of the swinepox virus. In the study, it has been demonstrated for the first time that nasal and oral swabs can be used for PCR diagnosis of the disease and for swinepox virus isolation. Phylogenetic analysis revealed that the isolated virus was closely related to SWPV isolates registered in Germany, USA, and Brazil, and slightly differed from the Indian isolates. During experimental infection of pigs, a low pathogenicity of the Russian isolate was observed. Our data provides the first report on the isolation and characterization of swinepox virus in Russia.

## 1. Introduction

Swinepox is a contagious general infection of pigs, characterized by the appearance of pox lesions on the skin of animals similar to smallpox lesions in humans [1]. At various times, the disease has been reported in different swine-raising countries, including Brazil, India, and European and North American countries, and it has been associated with economic losses [1,2,3,4,5,6,7,8,9,10,11,12,13,14]. It has been shown from previous research that the disease is age-related. In adult pigs, the disease usually occurs in a mild form with a lesion limited to the skin, while in young pigs (under the age of 4 months) the disease is severe, and the mortality rate for this condition is approaching 100% [3,15,16]. The most common signs of a poxvirus infection are characteristic skin lesions at the sites of penetration of the virus into the host body, mild fever, and inflammation of the local lymph nodes, while the usual general infection and viremia are not typically observed [17]. Infected pigs are the host reservoir, while hog lice (*Haematopinus suis)* and domestic flies (*Musca domestica)* serve as the mechanical vectors of infection. Infection in sows can be asymptomatic but can often lead to vertical transmission (mother-to-child), resulting in miscarriages and stillbirths [3,7,10].

The causative agent of swinepox is the SWPV virus of the genus *Suipoxvirus* of the *Poxviridae* family. This virus contains a linear double-stranded DNA genome with a length of 146 kbp and is the etiological agent of eruptive dermatitis in pigs. Viral DNA contains around 150 putative genes [8]. The penetration of the virus into the host cell is achieved by endocytosis after the attachment of the virus to the cell membrane. After that, the viral envelope membrane fuses with the cell membrane, and the virus then enters the cell.

Swine pox can be diagnosed by the appearance of typical clinical signs and lesions such as papules and pustules on the abdominal skin. Several molecular methods have also been developed to detect swine pox [9,18]. Recently, a one-step duplex PCR has been proposed for the detection of swinepox and vaccinia viruses in skin lesions of pigs with poxvirus-associated diseases. However, virus isolation is the gold standard for SWPV identification [19].

Outbreaks of SWPV disease are recorded sporadically in various regions of Russia. Since 2007, the virus has been detected in animals from 7 farms in four regions of Russia (Moscow, Kursk, Ryazan and Belgorod regions) [20]. However, the availability of data from epidemiological studies as well as data on the properties of the Russian SWPV isolates are extremely limited. In 2013, an outbreak of the disease with skin lesions was registered at a pig farm in the Belgorod region of Russia. The necessary quarantine measures were taken, as a result of which no other cases of swinepox infection have been registered in the region. This case was reported based on clinical and pathological findings and was later confirmed by virus isolation and molecular analysis.

The present article is the first report on the successful isolation of the swinepox virus in Russia. In this study, we also present the phylogenetic analysis of the virus that caused swinepox in the Belgorod region of the Russian Federation. It has been demonstrated for the first time that nasal and oral swabs can be used for PCR diagnosis of the disease and for virus isolation.

## 2. Materials and Methods

### 2.1. Sample Collection

In 2013, in the Belgorod region of Russia, on the territory of a pig-breeding complex, a disease associated with skin lesions was revealed among pigs. Swine pox was preliminarily diagnosed by clinical signs. Scab-like clinical specimens collected from the infected animals were transported to the Federal Research Center for Virology and Microbiology (FRCVM) for virus isolation and molecular detection of SWPV. The skin samples were stored at −20 °C.

### 2.2. Virus Isolation

Porcine kidney (PK-15) cells (PK15, ATCC CCL-33) were obtained from the American Type Culture Collection (ATCC, Manassas, VA, USA). The primary cell culture of porcine testicles was generated as described in [21]. Large white piglets aged from 1-day-old to 4-weeks-old were obtained from our own animal facility. Briefly, testicles from piglets were aseptically removed in accordance with standard approved procedures, placed in a 96 mm glass Petri dish on ice, and further processed in a laminar flow hood, where the testicles were minced into small pieces, washed, and then digested with 0.25% trypsin. The resulting cells were cultured in Eagle’s Minimal Essential Medium (MEM) (Gibco) containing 10% fetal bovine serum (Gibco), supplemented with an antimycotic antibiotic (Gibco), and were then maintained at 37 °C and 5% CO_2_. The complete medium was changed every 24 h for 4–6 days until the formation of a monolayer of cells.

For virus isolation, 70–80% confluent monolayers of cells (PK-15 and a primary cell culture of porcine testicles) in a T25 flask were inoculated with 1 mL of a clarified 10% skin scab suspension. After 60 min of adsorption, the viral material was removed, and 6 mL of fresh MEM was added. Cells were incubated for 7 days and observed daily under a microscope for the presence of a cytopathic effect (CPE). After incubation, the cells were frozen and thawed three times. The resulting clarified cell suspension was used for further passage of the virus and/or titration of virus. Five blind passages were performed in the cell culture to isolate the virus from the clinical samples.

To adapt the virus to the PK-15 cells, the 5th blind passage of the infected primary culture of porcine testes was used. The 70–80% confluent monolayers of the PK-15 cells in a T25 flask were inoculated with 1 mL of infected primary cell suspension. After 60 min of adsorption, viral material was removed and 5 mL of fresh MEM containing 2% fetal bovine serum was added. Cells were incubated for 7 days and observed daily under a microscope for the presence of CPE.

The virus was titrated by visualization of the CPE in the primary cell culture of porcine testicles. Titers were expressed as the tissue culture median infectious dose (TCID50), according to the Reed–Muench method [22].

### 2.3. DNA Isolation and Amplification

DNA was isolated from clinical specimens (skin scab) and from an infected porcine testicular cell culture using the DNeasy blood and tissue kit (Qiagen, Hilden, Germany) according to the manufacturer’s instructions. In-house real-time PCR was performed using a qPCRmix-HS kit (Evrogen, Moscow, Russia) with primers specific to the SPV032 gene (SPVU 5′-gtaссаttttggaggасаcg-3′, SPVD 5′-ttcaataaatcgccagttgtaс-3′, and Taqman probe SPVZ 5′-[FAM]ggtaccatatctatatatccctgttg[BHQ1]-3′, respectively). Cycling conditions were initial denaturation at 95 °C for 120 s, followed by 40 cycles of denaturation at 95 °C for 10 s, primer annealing, and finally extension at 60 °C for 20 s. 

PCR amplification of the viral late transcription factor-3 (VLTF-3) gene was performed as described by Medaglia et al. [9]. PCR was performed using ScreenMix-HS (Evrogen, Moscow, Russia) according to the manufacturer’s protocol. 

PCR amplification of the SPV119, SVP120, and SPV133 genes was conducted as previously described by Riyesh et al. [12] using ScreenMix-HS (Evrogen, Moscow, Russia). 

PCR products were analyzed by electrophoresis in 2% agarose gel in TAE buffer.

### 2.4. Phylogenetic Study

PCR products were purified using a Cleanup Standard kit (Evrogen, Moscow, Russia), and DNA was sequenced by the Sanger method on a 3130xl Genetic Analyzer (Thermo Fisher Scientific, Waltham, MA, USA), and then further analyzed using Sequence Analyzer software (Applied Biosystems, Waltham, MA, USA). The resulting viral genes were then aligned in MEGA-X using the ClustalW algorithm [23]. Phylogenetic trees were constructed using the maximum likelihood method, and the best-fit model was selected according to the ML analysis, being T92 + G + I [24,25]. Nodes were determined via bootstrap analysis with 500 replicates. Viral genes and genome segments were amplified by PCR and then sequenced using the first-generation Sanger DNA sequencing method [26]. 

### 2.5. Animal Experiment

A total of 15 large white pigs, 3 months old, and free from specific pathogens, were randomly divided into three groups (n = 5 per group). Each group was housed in the isolated rooms of the FRCVM animal facility for an acclimatization period of one week prior to infection as well as throughout the experiment (30 dpi). Pigs in group 1 were infected subcutaneously in 4 different areas of the neck and ears with a clarified 10% suspension of the skin crust, 1 mL per pig (0.25 mL per site). The pigs in group 2 were infected subcutaneously with the SWPV isolate adapted to PK-15 cells, 1 × 10^6^ TCID50 per piglet (0.25 × 10^6^ TCID50 per site). Pigs from group 3 were not infected and were kept as controls in the challenge experiment. 

After infection, all animals were examined daily for skin lesions and general clinical signs of SWP, and their rectal temperatures were also recorded. Clinical assessment of SPWV-infected animals was performed in 8 different categories (fever, anorexia, skin lesions, behavior, rhinitis, conjunctivitis, itching, and diarrhea, respectively) (Supplementary Materials Table S1). Blood samples, along with oral and nasal swabs were collected daily from day 3 to day 14 post infection (dpi), and then at 3-day intervals for detection of the virus and the SWPV genome.

## 3. Results

### 3.1. Virus Isolation and Adaptation

In order to isolate the virus, porcine kidney cells (PK-15) and a primary culture of pig testicles were inoculated with 1 mL of clarified 10% skin scab suspension. Unfortunately, it was not possible to isolate the virus from the clinical samples by culturing them on PK-15 cells, as CPEs were not observed in these cells during five consecutive blind passages. At the same time, the virus was successfully isolated using a primary culture of porcine testicular cells. During the first two passages, no visible changes in the cell culture were observed, and at the third blind passage, CPEs were characterized by the rounding and detachment of cells in certain sites of the cell monolayer. At the fifth blind passage, 3–4 days after infection (dpi), CPEs were observed, characterized by the massive rounding of cells, their detachment, and death, which is indicative an efficient viral replication in these cells. At the fifth passage, the virus accumulation was 6.00 ± 0.25 lg TCID50/mL. To adapt the SWPV isolate to laboratory cell cultures, the virus suspension from 5th passage was used to infect the PK-15 cells. Then, the virus was cultured in the PK-15 cell line for another 3–5 blind passages. Typical SWPV-induced CPEs (including cell rounding, increased granularity, degeneration, and monolayer detachment) were observed in the PK-15 cell at 6–7 dpi. Infectious activity was estimated at 6.5 ± 0.25 TCID50/mL, indicating effective virus replication in this cell line. The use of the continuous cell line PK-15 was deemed to be more convenient for the accumulation of large volumes of the virus, the production of recombinant SWPV strains, and the expression of foreign proteins in the case of using SWPV as a viral vector.

### 3.2. SWPV Identification

Two PCR assays were used to identify and confirm SWPV. First, an in-house real-time PCR was performed to amplify the SPV032 gene encoding a PKR inhibitor. All skin scab samples assessed by real-time PCR were SWPV-positive. To confirm these results, the viral late transcription factor-3 (VLTF-3) gene was amplified as described previously [9]. PCR amplification of the VLTF-3 gene fragment (482 bp) also showed that all scab samples from the affected animals were positive for SWPV. In addition, isolation of SWPV in primary culture cells of porcine testicles was confirmed by the PCR amplification of specific SWPV genes. Thus, the results of in-house real-time PCR were found to be correlated with the reference PCR amplification of the VLTF-3 gene, and the PCR proposed in this study could serve as a useful tool for the rapid specific detection of the swinepox virus.

### 3.3. Phylogenetic Study

For phylogenetic analysis of the Russian SWPV isolate, the sequences of the VLTF-3, SPV119, SVP120, and SPV133 genes were used. Amplified DNA fragments containing the indicated genes of the Russian SWPV isolate Alekseevskii were sequenced. The sequences of the VLTF-3, SPV119, SVP120, and SPV133 genes were deposited in GenBank under the accession numbers MW892733, MF045845.1, MF045846.1, and MF045847.1, respectively. Then, the gene sequences were aligned with the other known poxviruses sequences from GenBank. 

**Figure 1:**
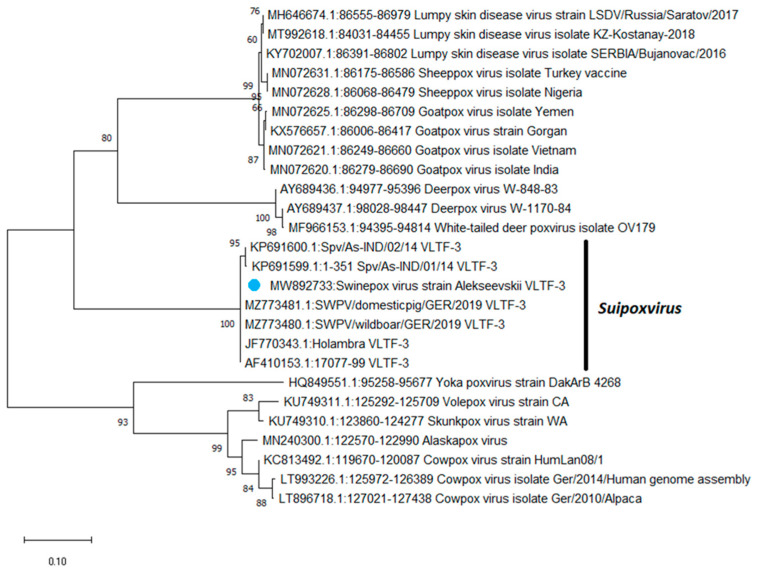
Phylogenetic tree showing the relationships of the various members of the *Poxviridae* family based on the sequences of the VLTF-3 gene. The phylogenetic tree was constructed using the maximum likelihood method. The percentage of replicated trees in which the associated taxa clustered together in the bootstrap test (500 replicates) with a percentage higher than 50 are shown next to the branches. The Russian SWPV isolate Alekseevskii reported in the Belgorod region in 2013 is indicated with a blue circle.

Based on the sequences of the VLTF-3 gene, a phylogenetic tree was constructed using the maximum likelihood method (Figure 1). As shown in Figure 1, based on the VLTF-3 gene sequences, swinepox viruses were grouped into a separate clade from the other poxviruses.

**Figure 2:**
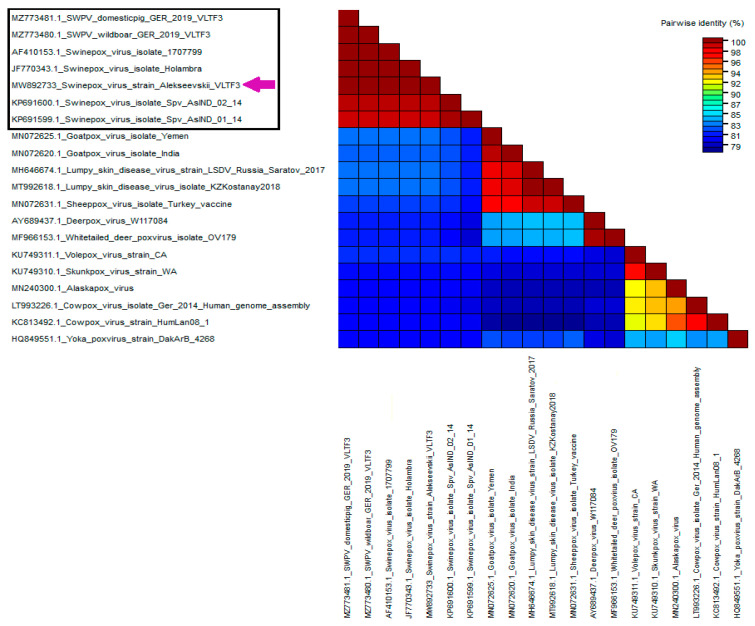
Pairwise nucleotide identity matrix of the Russian SWPV isolate Alekseevskii and other members of the *Poxviridae* family based on the VLTF-3 gene. The matrix was generated using SDT software v1.2. Members of the *Suipoxvirus* genus are boxed. The Alekseevskii isolate is indicated by a purple arrow.

Phylogenetic analysis based on VLTF-3 gene sequences revealed that the Russian isolate was grouped with SWPV isolates from the USA, Brazil, and Germany. Based on the identity matrix generated using the SDT 1.2 software, the Alekseevskii isolate showed a 100% identity with isolates 17077-99 (USA), Holambra (Brazil), SWPV/wildboar/GER/2019 (Germany), and SWPV/domestic/GER/2019 (Germany), and 98% identity with Spv/As-IND/01/14 (India) and Spv/As-IND/02/14 (India), respectively (Figure 2). At the same time, comparison of the VLTF-3 gene sequence of the Russian isolate with the sequences of the other poxviruses showed only an 82–83% identity (Figure 2).

For further genetic characterization of the Russian isolate, the amino acid sequences of the SPV119, SVP120, and SPV133 genes were compared with the sequences of the SWPV isolates from different geographic regions (USA, India, and Germany). It turned out that the Russian isolate Alekseevskii was 100% identical to isolates 17077-99 (USA), SWPV/wildboar/GER/2019 (Germany), and SWPV/domestic/GER/2019 (Germany) in the sequences of all three genes. However, comparison of the amino acid sequences of the SPV119, SPV120, and SPV133 genes of the Russian and Indian isolates revealed substitutions of individual amino acid residues. For these isolates, the amino acid sequence identity of SPV119, SPV120 and SPV133 was 96.75%, 97.05%, and 98.88%, respectively.

### 3.4. Animal Experiment

Two groups of pigs were infected subcutaneously in four different areas of the neck and ears. The first group was infected with a purified 10% suspension of skin scab, and the second with a suspension of the SWPV isolate adapted to PK-15 cells. Back titration of the material used to infect the animals of group 2 showed that the pigs were inoculated with material containing 1 × 10^6^ TCID50/mL, which matched the expected titer. The material used to the infect the animals of group 1 (clarified 10% skin scab suspension) was not titrated. Five pigs from group 3 (control group) were inoculated with sterile PBS (1 mL per pig) to mimic infection. 

**Figure 3:**
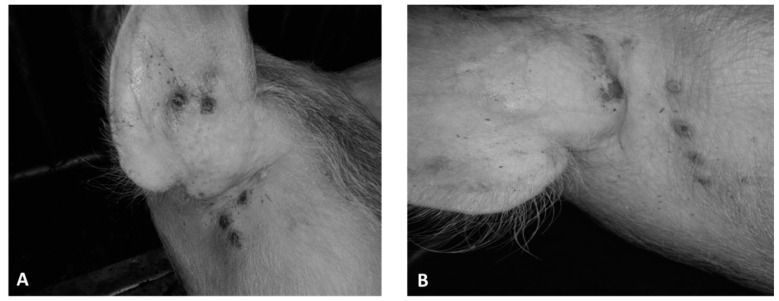
Vesicular skin lesions of pigs infected with the SWPV isolate Alekseevskii: (**A**) pig infected with a 10% clarified skin scab suspension, 6 dpi; and (**B**) pig infected with a virus adapted to PK-15 cells, 14 dpi, respectively.

**Figure 4:**
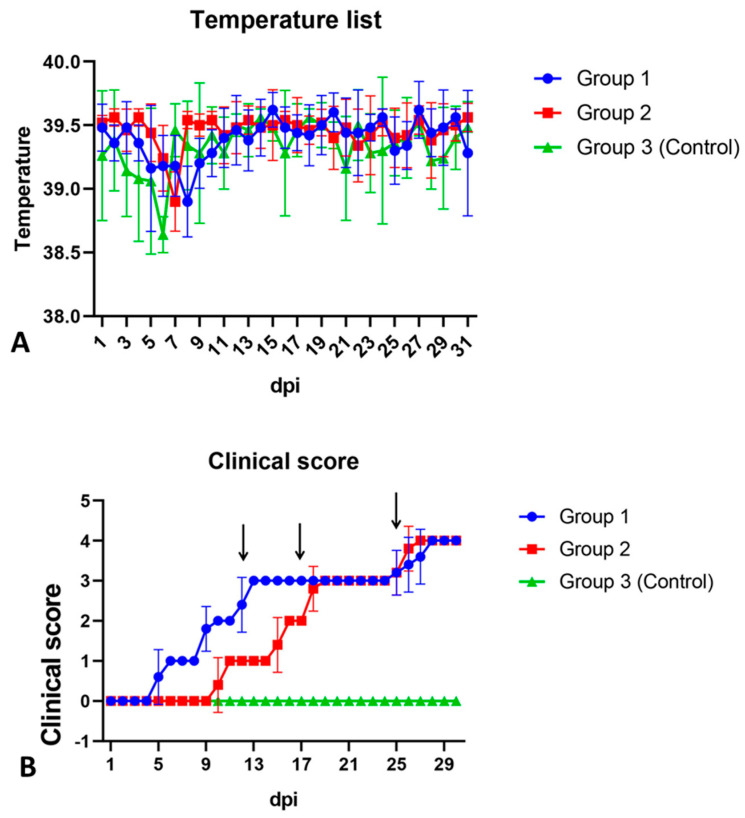
Group means of rectal temperature (**A**) and clinical score (**B**) with 95% confidence intervals for pigs inoculated with a purified 10% suspension of skin scab (blue line), the SWPV isolate Alekseevskii adapted to PK-15 cells (red line), and PBS (control group, green line). The points (dpi) at which the virus was isolated from the oral and nasal swabs samples are marked by an arrow. Analysis was conducted using Graphpad Prism software version 8.0.1.

Five days after infection, all animals in group 1 developed a vesicular skin lesion, which then turned into pustules (Figure 3A and Figure 4B). Animals of group 2 also showed similar vesicular lesions, which, however, appeared only 12–13 days post infection (Figure 3B). No other lesions or clinical signs of disease were observed in any of the animals, and rectal temperatures ranged from 38.5 to 39.6 °C (Figure 4A). All control pigs showed no clinical signs associated with the disease (Figure 4A,B). All animals of the three groups, regardless of gender and body weight, showed good appetite and activity until the end of the observation period (30 dpi).

Blood, along with oral and nasal swabs were collected daily from 3 to 14 days after infection (dpi), and then at 3-day intervals to detect the virus and SWPV genome. Real-time PCR analysis showed that all blood samples did not contain the viral DNA, indicating the absence of viremia in the infected animals. However, the presence of viral DNA was confirmed with PCR in oral and nasal swabs taken in the range of 8–11 to 30 dpi. Some samples with a positive PCR signal, collected on the 11th and 27th day after infection, were used for virus isolation in the primary culture of porcine testicles. The virus was successfully isolated from all selected samples after the third blind passage, in which the development of characteristic CPEs were observed. These results indicated the presence of an infectious virus in oral and nasal swab samples, and that the virus could be released into the environment over a sufficiently long period of time.

Information on the animal experiment is presented in Table 1.

## 4. Discussion

In 2013, in the Belgorod region of Russia, a disease associated with damage to the skin of pigs was detected on the territory of a pig breeding complex. Based on clinical signs, pathology, virus isolation, and molecular studies, a diagnosis of SWPV was made. Based on the negative PCR results, other viral pathogens were excluded as the cause of the disease. Unfortunately, there are no epidemiological data on which to estimate the overall morbidity, cumulative mortality, and mortality (case fatality rate, CFR) of this condition. The lack of these data made it impossible to state that adult pigs had also been sick during the outbreak on the farm to the same extent as the piglets. It is possible that the observed clinical signs that were detected only in piglets correlated with the results of other researchers who reported a higher morbidity and mortality of piglets due to the smallpox presence in pigsties [9,13,26,27]. In addition, parasites and secondary bacterial infection could have aggravated the condition of these animals and led to death, which was not the case with experimental infection. The results of a laboratory experiment using animals revealed a low pathogenicity of the Russian isolate for pigs. Indeed, the skin lesions mainly affected the ears and the area around the ears. In addition, no increases in temperature were observed in all affected animals during the experiment. Samples collected during other SWPV outbreaks in Russia showed the presence of secondary pathogens, such as *Streptococcus suis*, *Staphylococcus* spp., *Actinomyces pyogenes*, *Fusobacterium necrophorum*, and *Porcine circovirus* Type 2 (PCV2) [20]. It is possible that the presence of a secondary infection was the reason for the higher morbidity in pigs, which led to more pronounced clinical complications compared to our experimental infection.

To diagnose viral infections, it is customary to isolate the virus in cell cultures with subsequent molecular identification [28]. Isolation of the virus followed by immunofluorescent staining was presented as one of the most reliable methods for the laboratory diagnosis of swinepox [3,17,29]. However, today, this method is rarely used, since a faster and more sensitive method of molecular diagnostics based on PCR is generally used. In the present study, the isolation of the swinepox virus from clinical samples was conducted in a primary culture of porcine testis cells. Since SWPV and vaccinia virus (VACV) cause similar skin lesions, laboratory molecular testing was therefore needed to confirm the diagnosis. The PCR analysis of DNA isolated from these clinical samples (skin samples) and infected cell culture samples confirmed the presence of the SWPV genome. It has been demonstrated for the first time that nasal and oral swabs can be used for PCR diagnosis of disease and virus isolation. It is known that other poxviruses, such as sheep pox (SPV) and goat pox (GPS) viruses, are mainly found in oral, nasal, or ocular secretions [30,31]. Moreover, smears are recommended for diagnostic studies of these diseases due to the fact that in some studies, the sensitivity in smears was found to be higher compared to EDTA blood or serum [32].

In the present study, the isolated SWPV was successfully adapted to the PK-15 cell line. Although there are positive examples of the direct isolation of swinepox virus in the PK-15 cell culture from clinical samples [12,33], we failed to isolate the virus even after five blind passages of viral material. Therefore, for adaptation to PK-15, the material of the 5th passage of the virus was used in the primary culture of porcine testicles. The adapted virus replicated efficiently on the PK-15 cell line, showing specific cytopathic effects and infectious activity of at least 6 lg TCID/mL.

Recombinant poxviruses, including swinepox virus, are strong candidates as live viral vectors for use in humans and animals [34,35,36,37]. SWPV has several competitive advantages as a viral vector for pig immunization. It can be used as a primary vaccine as SPV induces cellular and humoral immune responses in porcine models [38]. There are several examples of the successful use of the virus to immunize pigs against various porcine pathogens [34,38,39,40,41]. SPV also exhibits a strong host specificity, only infecting pigs, although a wider host range has been reported in cultured cells [42]. Due to the low pathogenicity of the Russian isolate during the experimental infection of pigs, we assume that this isolate can be used in the future as a viral vector for the expression of foreign proteins. Although the Russian SWPV isolate adapted to PK-15 cells showed mild virulent characteristics, it was not fatal to pigs and did not spread to their organs. This isolate caused dermatological symptoms and was also found for a long time in both nasal and oral swabs. In addition, in this study, we did not consider a possible decrease in immunity in the infected animals. In this regard, we recommend that before being used in a veterinary clinic for immunization of pigs, this isolate should be additionally attenuated by removing the viral virulence factors (e.g., 003 and TK genes [38]) using genetic engineering.

Knowledge of the genetic diversity of strains, wildlife reservoirs, and the evolutionary origins of SWPV is limited. The sequences of the VLTF-3, SPV119, SVP120, and SPV133 genes were used for the phylogenetic analysis of the Russian isolate and the other SWPV isolates. The VLTF-3 gene encodes a viral late transcription factor and is often used in phylogenetic studies of SWPV. Two other genes, SPV119 and SPV120, encode extracellular envelope glycoproteins. It has been shown that the last gene, CPV133, encodes a protein belonging to the A52R-like family [43]. The A52 protein of another poxvirus, VACV, has been well studied, and has been shown to be a middle-early protein that contributes to SWPV virulence [44]. In addition, A52R was found to block IL-1 and TLR-mediated NF-kB activation [45]. Later, it was shown that VACV A52 interacts with IRAK-2, which promotes TRAF6 ubiquitination, which is necessary for NF-kB activation [46,47]. It is not yet clear whether the SWPV A52R-like family protein performs the same function as VACV A52, but it may be an important host-range protein that modulates the host response to infection. Phylogenetic analysis of the Russian isolate based on the sequences of the VLTF-3, SPV119, SVP120, and SPV133 genes revealed a 100% identity of the Alekseevskii isolate with isolates 17077 (USA), SWPV/wildboar/GER/2019 (Germany) and SWPV/domestic/GER/2019 (Germany), respectively. At the same time, the Russian isolate, unlike the Indian ones, contained single amino acid substitutions, and the 100% sequence identity of several genes of the Russian isolate and the German SWP viruses isolated in 2019–2020 may indicate the genetic relationship with the European isolate. The persistence of SPWV in wild boar populations in Germany [14] may also point to possible reservoirs of the virus in wildlife and transmission routes in European countries. In this regard, it is of great interest to analyze the persistence of this pathogen in the wild boar population in Russia.

The last outbreak of swinepox registered in Russia occurred in 2019 in the Kaluga region. This region borders the Belgorod region, which may indicate the persistence of the virus in this area. Unfortunately, we did not receive material from the Kaluga region for the analysis of this isolate. This information could help in further studying the epidemiology of swine pox in Russia, and in determining whether new outbreaks are caused by isolates circulating in the country or isolates imported from abroad. Further studies of genome-wide Russian SPWV isolates are essential to determine their sequence diversity and draw conclusions about the transmission routes and possible reservoirs of the virus.

## 5. Conclusions

In conclusion, this is the first study describing the successful isolation and characterization of SWPV in Russia. The presence of SWPV in the scab samples was evaluated by in-house real-time PCR, reference PCR amplification, and nucleotide sequencing of the viral late transcription factor-3 (VLTF-3) gene and was then confirmed by virus isolation. Phylogenetic analysis based on VLTF-3 gene sequences revealed that the Russian isolate was grouped with SWPV isolates from the USA, Brazil, and Germany. Due to the low pathogenicity of the Russian isolate during the experimental infection of pigs, we assume that this isolate can be used following genetic modification in the future as a viral vector for the expression of foreign proteins or recombinant vaccine development. In addition, it has been demonstrated that the real-time PCR proposed in this study can serve as a useful tool for the rapid specific detection of the swinepox virus.

## Figures and Tables

**Table 1:** Animal experiment data on pig survival, fever, SPW skin lesion, results of PCR, and SPW virus isolation.

Group	No. of Animals	Infection Material	Survival %	Fever	SPW Skin Lesion	Viremia	SWPV DNA in Oral and Nasal Swabs	SWPV in Oral and Nasal Swabs
1	5	Clarified 10% suspension of skin scab (not titrated)	100	No	Yes at 5–30 dpi	No	Yes at 8–30 dpi	Yes, isolated at 11, 17, and 25 dpi
2	5	Adapted to PK-15 cells SWPV isolate (1 × 10^6^ TCID50)	100	No	Yes at 11–30 dpi	No	Yes at 11–30 dpi	Yes, isolated at 11, 17, and 25 dpi
3	5	PBS	100	No	No	No	No	No

## Data Availability

The sequences of VLTF-3, SPV119, SVP120, and SPV133 genes were deposited in GenBank under the accession numbers MW892733, MF045845.1, MF045846.1, and MF045847.1, respectively.

## Supplementary Materials

The following supporting information can be downloaded at: https://www.mdpi.com/article/10.3390/ani13111786/s1. Table S1: Clinical Scoring of pigs inoculated with swinepox virus.
