# Supplementary material for: Isolation and Characterization of Swinepox Virus from Outbreak in Russia
Source: Animals (Basel). 2023 May 27;13(11):1786. doi: 10.3390/ani13111786 (PMC10252027; doi:10.3390/ani13111786)
Supplement: Supplementary file 1 [file animals-13-01786-s001.zip › animals-2382264-supplementary.pdf]

Supplementary Materials

Table S1. Clinical Scoring of pigs inoculated with swinepox virus.

CHARACTERISTIC	SCORE (POINTS)	
**1. ANOREXIA**	0	Without changes
	1	Reduced eating
	2	Partial refusal of feed
	3	Not eating
**2. BEHAVIOR**		
	0	Without changes
	1	Depressed
	2	Gets up only slowly when touched
	3	Remains recumbent when touched
**3. SKIN LESIONS**		
	0	Normal
	1	Exanthema
	2	Papules
	3	Suppuration
	4	Scarring
**4. RHINITIS**		
	0	Normal
	1	Attended
**5. CONJUNCTIVITIS**		
	0	Normal
	1	Attended
**6. BODY TEMPERATURE**		
	0	38.0-39.9
	1	40.0-41.0
	2	41.1-42.0
**7. ITCHING**		
	0	Normal
	1	Attended
**8. DIARRHEA**		
	0	Normal
	1	Watery diarrhea
	2	Severe bloody diarrhea
